# Stool carriage of CTX-M/CMY-producing *Salmonella enterica* in a Chinese tertiary hospital in Shenzhen, China

**DOI:** 10.3389/fcimb.2025.1544757

**Published:** 2025-03-13

**Authors:** Jing Wang, Zi-Han Dong, Xian-Yuan Zhou, Qin-Chun Ma, Zhen-Yu Wang, Dachuan Lin, Ying-Feng Huang, Chi Zhang, Xinan Jiao, Deng Li, Qiuchun Li

**Affiliations:** ^1^ Jiangsu Key Laboratory of Zoonosis/Jiangsu Co-Innovation Center for Prevention and Control of Important Animal Infectious Diseases and Zoonoses, Yangzhou University, Yangzhou, China; ^2^ Institute of Medical Sciences, School of Public Health, Xinjiang Medical University, Urumqi, China; ^3^ Department of Laboratory Medicine, Shenzhen University General Hospital, Shenzhen, China; ^4^ Key Laboratory of Tropical Translational Medicine of Ministry of Education, Hainan Medical University-The University of Hong Kong Joint Laboratory of Tropical Infectious Diseases, School of Basic Medicine and Life Sciences, Hainan Medical University, Haikou, China

**Keywords:** non-typhoidal *Salmonella*, *S.* 4, [5], 12:i:-, *S.* Enteritidis, *S.* Typhimurium, *bla* ^CTX-M^, Typhimurium monophasic variant S. 4

## Abstract

Salmonellosis, caused by non-typhoidal *Salmonella*, is a common foodborne gastrointestinal infection. Third-generation cephalosporins are recommended as the first-line treatment for *Salmonella* infections. Our study aimed to investigate the molecular epidemiology, antimicrobial resistance, and the transmission of extended-spectrum β-lactamases (ESBL) genes in 96 clinical *Salmonella* isolates collected between 2020 and 2022 at a tertiary hospital in Shenzhen, China. We performed antimicrobial susceptibility testing and whole-genome sequencing to identify serotypes, multilocus sequence typing, antimicrobial resistance genes in these isolates, and the genetic structures of the *bla*_CTX-M_/*bla*_CMY_ genes. Seventeen *Salmonella* serotypes were identified, with *S*. 4,[5],12:i:- (37.5%) being the most common, followed by *S*. Enteritidis (15.63%), *S*. Typhimurium (14.58%), *S.* London (7.29%), and *S.* Rissen (5.21%). MLST analysis revealed 19 distinct sequence types (STs), with ST34 being the most prevalent (36.46%), followed by ST11 (15.63%) and ST19 (13.54%). Antimicrobial resistance testing showed those isolates had high levels of resistance to ampicillin (72.92%) and tetracycline (71.88%), with 70.83% of isolates as multidrug-resistant (MDR). Three *bla*_CTX-M_ genes (*bla*_CTX-M-14,_ *bla*_CTX-M-55_, and *bla*_CTX-M-65_) and *bla*_CMY-2_ were identified among 18 cefotaxime-resistant strains, of which one and 12 isolates successfully transferred *bla*_CMY_ or *bla*_CTX-M_ to *E. coli* C600 via conjugation, respectively. The *bla*_CTX-M_/*bla*_CMY-2_-carrying contigs in nine *Salmonella* isolates ranged from 2,156 to 164,862 bp, were located either on the chromosome (n=1) or plasmids (IncI1, IncK1, IncA/C) (n=9), and the *bla*_CTX-M_/*bla*_CMY-2_ genes were associated with IS*Ecp1*. Our study demonstrates the diversity of MDR *Salmonella* serotypes in clinical isolates, and highlights the role of plasmids and mobile genetic elements in the horizontal transfer of *bla*_CTX-M_/*bla*_CMY_, emphasizing the need for continuous surveillance of *Salmonella* in clinical samples.

## Introduction

Non-typhoidal *Salmonella* is a leading cause of foodborne illness globally, causing salmonellosis, which typically presents with symptoms such as diarrhea, fever, abdominal cramps, and vomiting (CDC, 2019; EFSA and ECDC, 2023). In severe cases, particularly in immunocompromised individuals, *Salmonella* infection can lead to life-threatening complications (Ruiz et al., 2004). These infections are primarily caused by the consumption of *Salmonella*-contaminated food, particularly raw or undercooked poultry, eggs, and beef (EFSA and ECDC, 2023). To date, 2,659 *Salmonella* serovars have been identified (Monte and Sellera, 2020), with *Salmonella enterica* serovar Typhimurium (including its monophasic variant) and *S.* Enteritidis being the most common serotypes in human infections (EFSA and ECDC, 2023).

In recent decades, antimicrobial resistance has become a significant challenge in treating *Salmonella* infections (CDC, 2019; EFSA and ECDC, 2023). Third-generation cephalosporins are considered first-line antibiotics for treating *Salmonella* infection, but the isolates acquiring genes to produce extended-spectrum β-lactamases (ESBLs) - enzymes can confer the bacterial resistance to a broad range of β-lactam antibiotics, including penicillins and cephalosporins, which has significantly restricted treatment options (Ruiz et al., 2004; Crump et al., 2015; Bevan et al., 2017). The global prevalence of ESBL-producing *Salmonella*, particularly strains encoding CTX-M/CMY β-lactamases, continues to rise, presenting an increasing public health concern worldwide (Sun et al., 2022; Wang et al., 2020). Therefore, monitoring these *Salmonella* strains is crucial for understanding their prevalence and transmission characteristics. This study aims to investigate the molecular epidemiology and antimicrobial resistance characteristics of *Salmonella* isolates, with a particular focus on the prevalence and transmission of ESBL-producing strains in a tertiary hospital in Shenzhen, China.

## Materials and methods

### Bacterial isolates

From March 2020 to November 2022, 96 *Salmonella* strains were isolated from non-repetitive samples, including feces (n=93), blood (n=2), and purulent secretion (n=1), obtained from 87 different patients for routine diagnostics at a tertiary hospital in Shenzhen, China. The collected samples (2-3 g for feces; 1-3 mL for other liquid samples) were inoculated onto Salmonella Shigella (SS) agar plates using a standardized streaking technique. The plates were then incubated aerobically at 37°C for 24 hours. Following incubation, a single presumptive *Salmonella* colony was selected from each plate and subjected to species identification using matrix-assisted laser desorption/ionization time-of-flight mass spectrometry. Colonies confirmed as *Salmonella* spp. were subsequently streaked onto xylose lysine deoxycholate (XLD) agar plates for purification.

### Antimicrobial susceptibility testing

All *Salmonella* isolates were tested for susceptibility to colistin using the ISO-standard broth microdilution method, recommended by the joint CLSI-EUCAST Polymyxin Breakpoints Working Group (https://www.eucast.org/fileadmin/src/media/PDFs/EUCAST_files/General_documents/Recommendations_for_MIC_determination_of_colistin_March_2016.pdf). In addition, susceptibility to 12 other antimicrobial agents, including ampicillin, cefotaxime, meropenem, gentamicin, amikacin, streptomycin, tetracycline, chloramphenicol, nalidixic acid, ciprofloxacin, fosfomycin, and sulfamethoxazole/trimethoprim, was assessed using the agar dilution method following the guidelines of the Clinical and Laboratory Standards Institute (CLSI) M07 (CLSI, 2012). *Escherichia coli* ATCC 25922 was used as the quality control strain. Results were interpreted according to the 32^nd^ edition of the CLSI M100 (CLSI, 2022). The interpretation of streptomycin (>16 mg/L) was based on the epidemiological cut-off value for *Salmonella enterica* established by the European Committee on Antimicrobial Susceptibility Testing (EUCAST; www.eucast.org).

### Whole genome sequencing and analysis

Genomic DNA from all *Salmonella* isolates was extracted using the TIANamp Bacteria DNA Kit (Tiangen, Beijing, China) and sequenced on the Illumina NovaSeq platform. The genomic libraries were prepared using the NEB NEXT Ultra DNA Library Prep Kit for Illumina (New England Biolabs, USA), and sequencing was performed to generate 150 bp paired-end reads. For each *Salmonella* isolates subjected to WGS, a minimum coverage depth of 100× was achieved. Raw reads with less than 90% Q30 bases were trimmed and filtered using the NGSQC Toolkit v2.3.3, and assembled into contigs using SPAdes 3.8.2 (Bankevich et al., 2012). Serotypes were determined using the *Salmonella In Silico* Typing Resource (SISTR) (Yoshida et al., 2016). Genomic sequences were subjected to multilocus sequence typing (MLST) analysis using MLST 2.0 (https://cge.food.dtu.dk/services/MLST/). Additionally, antimicrobial resistance genes and mutations were identified using ResFinder and PointFinder, respectively (http://genepi.food.dtu.dk/resfinder). Contigs carrying *bla*_CTX-M_/*bla*_CMY_ genes were retrieved from the draft genomes and analyzed using the ISfinder platform (https://www-is.biotoul.fr) to identify insertion sequences, PlasmidFinder 2.1 (https://cge.food.dtu.dk/services/PlasmidFinder/) to detect plasmid replicons, and BLAST (https://blast.ncbi.nlm.nih.gov/Blast.cgi) for sequence homology and annotation.

### Conjugation experiments

Conjugation experiments were performed using cefotaxime-resistant *Salmonella* strains as donors and *E. coli* C600 (which exhibits high-level streptomycin resistance) as the recipient. Donor and recipient strains were inoculated in 2 mL of LB broth and incubated at 37°C, 180 rpm for 4 hours. The cultures were then mixed in a 1:4 ratio (v/v) and incubated at 37°C for 12 h. Transconjugants were selected on MacConkey agar plates containing cefotaxime (2 mg/mL) and streptomycin (3000 mg/mL) and confirmed by PCR detection of *bla*_CTX-M_ or *bla*_CMY_ (Liu et al., 2007). All experiments were conducted in triplicate.

### Nucleotide sequence accession number

The whole genome sequences of the *Salmonella* isolates have been deposited in GenBank under the accession number: PRJEB83553.

## Results

### Distribution of *Salmonella* isolates

**Figure 1:**
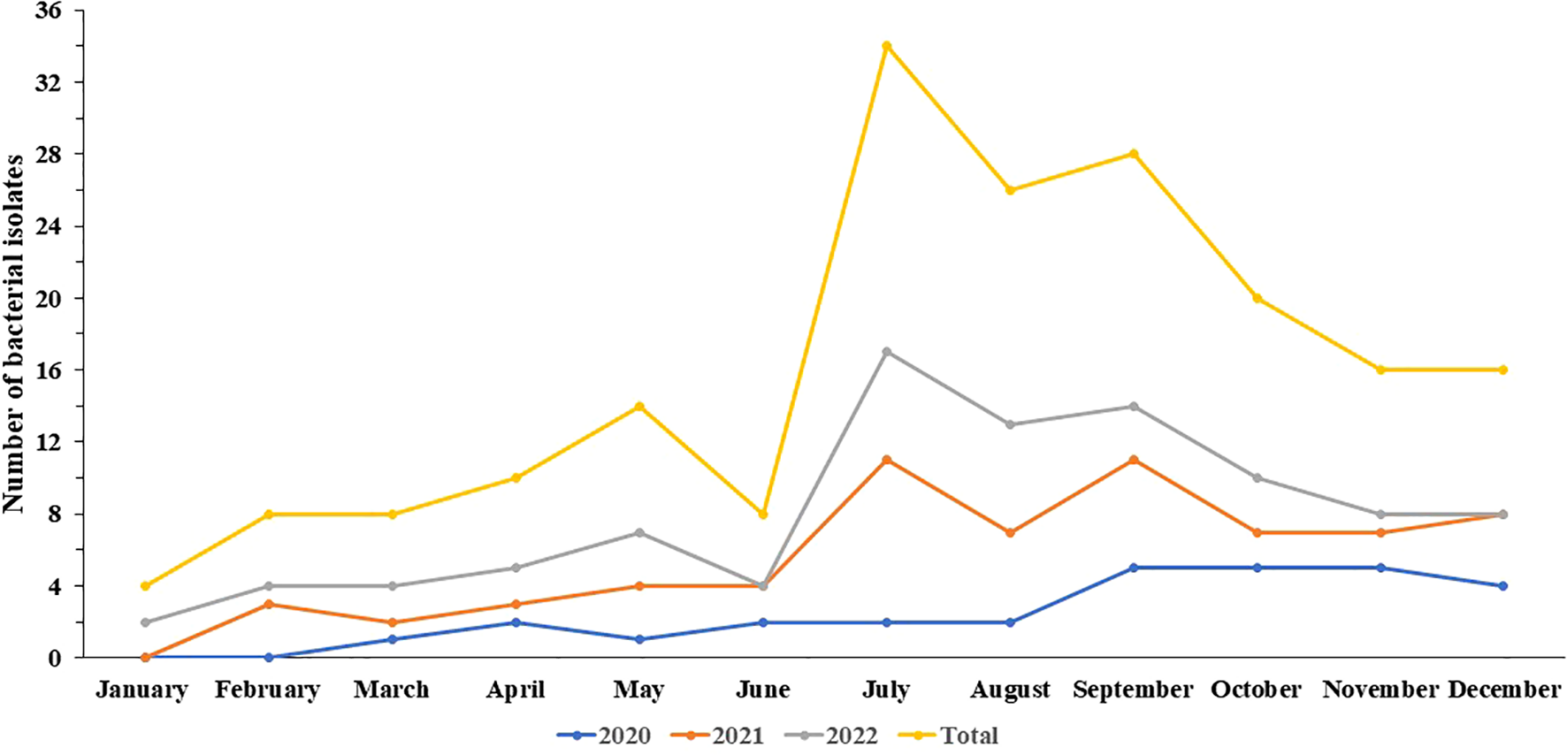
The trend of the incidences of 96 *Salmonella* isolates in this study.

This study analyzed 96 *Salmonella* isolates obtained from fecal (96.88%, n=93), blood (2.08%, 2/96), and pus samples (1.04%, 1/96) from 87 patients. Of these, 29 isolates were collected in 2020, 38 in 2021, and 29 in 2022, representing 30.21%, 39.58%, and 30.21%, respectively. As shown in **Figure 1**, the highest detection rate occurred in July (17.71%, n=17), while the lowest was observed in January (2.08%, n=2). The patient cohort comprised 53 males and 34 females, with a male-to-female ratio of 1.56:1. The age of the patients ranged from 24 days to 80 years, with 77.01% (n=67) aged ≤ 5 years, 20.69% (n=18) between 6 and 59 years old, and 2.30% (n=2) ≥ 60 years.

### Serotypes and MLST of *Salmonella* isolates

**Figure 2:**
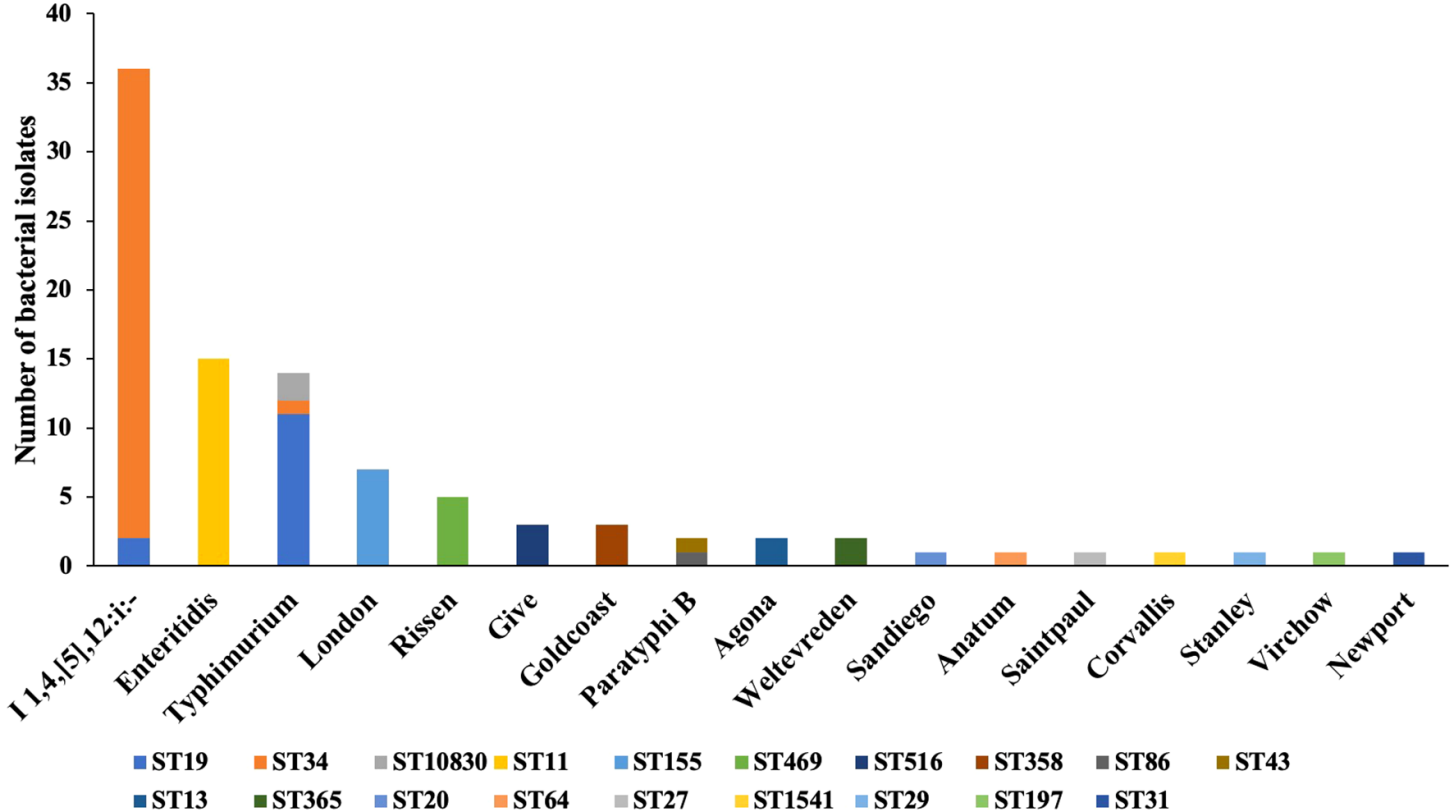
The prevalence of individual serovar with their sequence type (ST) detected in this study.

A total of 17 different *Salmonella* serotypes were identified among the 96 isolates (**Figure 2**). The most common serotype was *S.* Typhimurium monophasic variant *S*. 4,[5],12:i:- (37.50%, n=36), followed by *S.* Enteritidis (15.63%, n=15), *S.* Typhimurium (14.58%, n=14), *S.* London (7.29%, n=7), and *S.* Rissen (5.21%, n=5). *S*. Give and *S*. Goldcoast each accounted for 3.13% (n=3), while *S*. Paratyphi B, *S*. Agona, and *S*. Weltevreden each had two isolates. Additionally, one isolate was identified for each of the following serovars: *S*. Sandiego, *S*. Anatum, *S*. Saintpaul, *S*. Corvallis, *S*. Stanley, *S*. Virchow, and *S*. Newport.

The 96 *Salmonella* isolates were classified into 19 sequence types (STs) using *in silico* MLST (**Figure 2**). The most prevalent ST was ST34 (36.46%, n=35), followed by ST11 (15.63%, n=15), ST19 (13.54%, n=13), ST155 (7.29%, n=7), and ST469 (5.21%, n=5). A novel ST, named ST10830, was identified in two *S*. Typhimurium isolates and differed from ST34 in the *purE* locus.

Comparison of MLST and serotyping revealed that each ST generally corresponds to a single serotype, with the exceptions of ST19 and ST34. Specifically, ST19 included two isolates of *S*. 4,[5],12:i:- and 11 isolates of *S.* Typhimurium, while ST34 comprised 34 isolates of *S*. 4,[5],12:i:- and one of *S.* Typhimurium. Additionally, three serovars were associated with multiple STs: 36 *S*. 4,[5],12:i:-isolates included both ST19 and ST34; 14 *S.* Typhimurium isolates included ST19, ST34 and ST10830; and two *S*. Paratyphi B isolates were classified as ST43 and ST86.

### Antimicrobial resistance phenotypes and genotypes of *Salmonella* isolates

Antimicrobial susceptibility testing of the 96 *Salmonella* isolates revealed high resistance rates to ampicillin (72.92%, n=70) and tetracycline (71.88%, n=69), followed by streptomycin (48.96%, n=47), sulfamethoxazole/trimethoprim (44.79%, n=43), and chloramphenicol (43.75%, n=42). Additionally, gentamicin resistance was observed in 15 (15.63%) isolates. One *S*. 4,[5],12:i:- isolate (1.04%) from a one-year-old male infant showed resistance to amikacin and fosfomycin, while four *S.* Enteritidis isolates (4.17%) were resistant to colistin. All isolates were susceptible to meropenem. Additionally, 18 isolates (18.75%) exhibited resistance to cefotaxime, while 28 (29.17%) and three (3.13%) isolates were resistant to nalidixic acid and ciprofloxacin, respectively. Particularly, only one isolate (1.04%) exhibited co-resistance to third-generation cephalosporin (cefotaxime) and fluoroquinolone (ciprofloxacin), which are considered first-line treatments for salmonellosis.

Of the 96 *Salmonella* isolates, seven (7.29%) were pan-susceptible, exhibiting susceptibility to all tested antimicrobial agents, while 68 isolates (70.83%) were resistant to at least three antimicrobial classes and thus classified as multidrug-resistant (MDR). Among the 89 isolates resistant to at least one antimicrobial agent, 34 distinct antimicrobial resistance patterns were identified. The most common pattern was resistance to ampicillin-tetracycline-chloramphenicol-sulfamethoxazole/trimethoprim, observed in 13 isolates (13.54%) (**Supplementary Table S1**).

**Figure 3:**
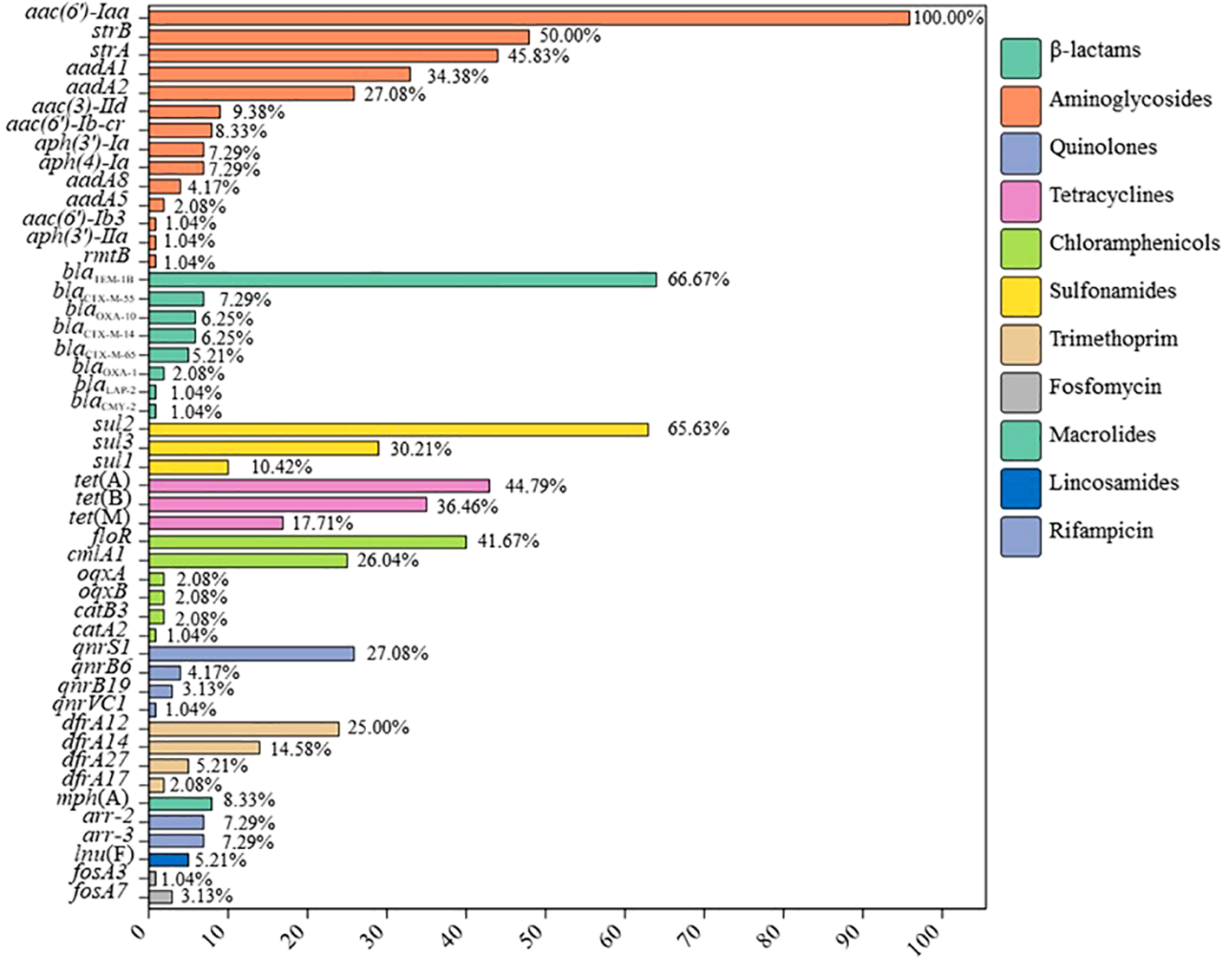
The prevalence of resistance genes in the 96 *Salmonella* isolates in this study.

To investigate the genetic mechanisms underlying antimicrobial resistance, whole-genome sequences of all isolates were screened for resistance genes and mutations in the quinolone resistance-determining region (QRDR). A total of 48 different resistance genes were identified across the 96 *Salmonella* isolates, conferring resistance or reduced susceptibility to β-lactams, aminoglycosides, quinolones, tetracyclines, chloramphenicols, sulfonamides, trimethoprims, fosfomycin, macrolides, lincosamides, and rifampicin (**Figure 3**). Each *Salmonella* isolate carried one to 21 resistances genes. They all carried cryptic aminoglycosides resistance gene *aac(6’)-Iaa* located on the chromosome. Additionally, one *S*. Sandiego and two *S*. Agona strains carried the chromosomal silent fosfomycin resistance gene *fosA7*. The amikacin- and fosfomycin- resistant *S*. 4,[5],12:i:- isolate carried the *rmtB* and *fosA3* genes. Eighteen cefotaxime-resistant isolates carried either *bla*_CTX-M_ (n=18) or *bla*_CMY-2_ (n=1). Three *bla*_CTX-M_ variants were detected: *bla*_CTX-M-14_ (n=6), *bla*_CTX-M-55_ (n=7), and *bla*_CTX-M-65_ (n=5). One *S*. Agona isolate harbored both *bla*_CTX-M-14_ and *bla*_CTX-M-55_. Other β-lactamase genes were also detected, including *bla*_TEM-1_ (n=64), *bla*_LAP-2_ (n=1), *bla*_OXA-1_ (n=2), and *bla*_OXA-10_ (n=6). Thirty-seven *Salmonella* strains harbored one or two quinolone resistance genes, including *qnrS1* (n=26), *qnrB6* (n=4), *qnrB19* (n=3), *qnrVC1* (n=1), *oqxAB* (n=2), and *aac(6’)-Ib-cr* (n=8). Furthermore, 13 *S*. Enteritidis isolates contained a single mutation in *gyrA* (D87G or D87Y), and five *S*. 4,[5],12:i:- strains and three *S.* Typhimurium strains also had a single mutation in *gyrA* (D87G, D87Y, or S83F). The *gyrA* mutation, along with the presence of quinolone resistance genes, contributed to resistance to nalidixic acid and ciprofloxacin in two *S*. 4,[5],12:i:- strains and one *S.* Typhimurium strain. The remaining 25 nalidixic acid-resistant isolates carried a *gyrA* mutation (n=18) or one quinolone resistance gene (*qnrB19*; n=3) or two quinolone resistance genes (*qnrB6* and *aac(6’)-Ib-cr*; n=4).

### Characterization of cefotaxime-resistant ESBLs/AmpC-producing *Salmonella* isolates

As shown in **Table 1**, 18 cefotaxime-resistant *Salmonella* isolates were collected from fecal samples, 15 of which were from patients aged less than 5 years. These isolates included nine *S*. 4,[5],12:i:- (ST34), three *S*. Enteritidis (ST11), two *S*. Agona (ST13), one *S*. Typhimurium (ST19), one *S*. Saintpaul (ST27), one *S*. Paratyphi B (ST86), and one *S*. Weltevreden (ST365). Notably, all three *S*. Enteritidis isolates carried *bla*_CTX-M-14_, and *bla*_CTX-M-65_ was identified exclusively in *S*. 4,[5],12:i:- strains. All ESBLs/AmpC-producing isolates exhibited resistance to ampicillin (MIC>128 mg/L) and had cefotaxime MICs ranging from 32 to >128 mg/L. Except for isolates SZ21HS63 and SZ22HS72, the remaining cefotaxime-resistant isolates were also resistant to multiple antibiotics. Thirteen of these isolates successfully transfer cefotaxime resistance to *E. coli* C600 via conjugation (**Table 1**). In addition to *bla*_CTX-M_/*bla*_CMY_, the isolates carried one to 20 other resistance genes, such as *bla*_TEM_, *bla*_OXA_, *strA*, *strB*, *tet*(A), *qnrS1*, *sul2*, and *fosA3* (**Table 1**). Additionally, mutations within *gyrA* (D87Y or D87G) were identified in four isolates (**Table 1**). Interestingly, two *S*. 4,[5],12:i:- isolates (SZ20HS5 and SZ20HS17) and two *S*. Enteritidis isolates (SZ20HS10 and SZ20HS18) were obtained from the same infants sampled at different time points, respectively, sharing identical STs, resistance profiles, resistance genes, and mutations.

**Table 1:** Chracterization of cefotaxime-resistant *Salmonella* isolates in this study.

Strains^a^	serotype	MLST	Source^b^	Sampling Date	ESBL genotype	Other resistance genes	Antimicrobial susceptibility profile^c^	Mutation^d^	Location of *bla*_CTX/CMY_ ^e^	Length of *bla*_CTX/CMY_-contig (bp)
SZ20HS5*	*S*. 1,4,[5],12:i:-	34	M, 11m	2020.09.18	*bla*_CTX-M-65_	*bla*_OXA-10_/*bla*_TEM-1B_/*aac(6’)-Iaa*/*aadA1*/*strA*/*strB*/ *tet*(A)/*tet*(B)/*cmlA1*/*floR*/*qnrS1*/*sul2*/*dfrA14*/*arr-2*	AMP/CTX/STR/TET/CHL/FFC/SXT	N	ND	5,837
SZ20HS10*	*S*. Enteritidis	11	M, 1y	2020.10.10	*bla*_CTX-M-14_	*bla*_TEM-1B_/*aac(6’)-Iaa*/*strA*/*strB*/*tet*(A)/*sul2*	AMP/CTX/STR/TET/NAL/CL	*gyrA* (D87Y)	IncI1	92,725
SZ20HS12	*S*. Saintpaul	27	F, 1y	2020.10.14	*bla*_CTX-M-55_	*bla*_TEM-1B_/*bla*_LAP-2_/*aac(6’)-Iaa*/*aph(3’)-Ia*/*strB*/*tet*(A)/*floR*/*qnrS1*/*dfrA14*/*mph*(A)/*arr-2*	AMP/CTX/TET/CHL/FFC	N	ND	7,178
SZ20HS17	*S*. 1,4,[5],12:i:-	34	M, 1y	2020.11.04	*bla*_CTX-M-65_	*bla*_OXA-10_/*bla*_TEM-1B_/*aac(6’)-Iaa*/*aadA1*/ *strA*/*strB*/*tet*(A)/*tet*(B)/*cmlA1*/*floR*/*qnrS1*/*sul2*/ *dfrA14*/*arr-2*	AMP/CTX/STR/TET/CHL/FFC/SXT	N	ND	5,837
SZ20HS18*	*S*. Enteritidis	11	M, 1y	2020.11.04	*bla*_CTX-M-14_	*bla*_TEM-1B_/*aac(6’)-Iaa*/*strA*/*strB*/*tet*(A)/*sul2*	AMP/CTX/STR/TET/NAL/CL	*gyrA* (D87Y)	IncI1	92,725
SZ20HS19*	*S*. Enteritidis	11	M, 1y	2020.11.04	*bla*_CTX-M-14_	*bla*_TEM-1B_/*aac(6’)-Iaa*/*strA*/*strB*/*tet*(A)/*sul2*	AMP/CTX/STR/TET/NAL	*gyrA* (D87Y)	IncI1	92,219
SZ21HS32*	*S*. 1,4,[5],12:i:-	34	M, 1y	2021.05.28	*bla*_CTX-M-65_	*bla*_OXA-10_/*bla*_TEM-1B_/*aac(6’)-Iaa*/*aph(3’)-Ia*/*aph(4)-Ia*/*aac(3)-IV*/*aadA1*/*aadA2*/*strA*/*strB*/*tet*(A)/*tet*(B)/*cmlA1*/*floR*/*qnrS1*/*sul2*/*sul3*/*dfrA14*/*arr-2*/*lnu*(F)	AMP/CTX/GEN/STR/TET/CHL/FFC/NAL/CIP/SXT	*gyrA* (D87G)	ND	5,561
SZ21HS33*	*S*. 1,4,[5],12:i:-	34	M, 3y	2021.05.28	*bla*_CMY-2_	*bla*_OXA-10_/*bla*_TEM-1B_/*aac(6’)-Iaa*/*aadA1*/*aac(6’)-Ib3*/*strA*/*strB*/*tet*(A)/*tet*(B)/*floR*/*qnrVC1*/*aac(6’)-Ib-cr*/*sul2*/*dfrA14*/*mph*(A)	AMP/CTX/STR/TET/CHL/FFC/SXT	N	IncA/C	92,168
SZ21HS34*	*S*. Agona	13	F, 4y	2021.06.01	*bla*_CTX-M-14/_ *bla*_CTX-M-55_	*bla*_TEM-1B_/*aac(6’)-Iaa*/*aac(3)-IId*/*strA*/*strB*/*tet*(A)/*floR*/*qnrS1*/*fosA7*/*sul2*/*sul3*/*dfrA14*	AMP/CTX/GEN/STR/TET/CHL/FFC/SXT	N	ND /chromosome	3,113 /22,529
SZ21HS55*	*S*. 1,4,[5],12:i:-	34	M, 1y	2021.09.08	*bla*_CTX-M-55_	*bla*_TEM-1B_/*aac(6’)-Iaa*/*aph(3’)-IIa*/*strA*/*strB*/*rmtB*/*tet*(B)/*fosA3*/*sul2*	AMP/CTX/GEN/AMI/STR/TET/FOS	N	ND	2,156
SZ21HS63*	*S*. Paratyphi B	86	M, 38y	2021.11.03	*bla*_CTX-M-14_	*aac(6’)-Iaa*/*sul2*	AMP/CTX	N	IncI1	96,781
SZ21HS66*	*S*. Typhimurium	19	M, 51y	2021.12.14	*bla*_CTX-M-55_	*bla*_TEM-1B_ */aac(6’)-Iaa/aadA2*/*tet*(A)/*tet*(M)/*cmlA1/floR*/*qnrS1*/*sul2*/*sul3*/*dfrA12*	AMP/CTX/TET/CHL/FFC/SXT	N	IncI1	84,817
SZ22HS72*	*S*. Weltevreden	365	F, 3y	2022.01.20	*bla*_CTX-M-14_	*aac(6’)-Iaa*	AMP/CTX	N	IncK	92,206
SZ22HS73	*S*. 1,4,[5],12:i:-	34	F, 2y	2022.02.09	*bla*_CTX-M-55_	*bla*_TEM-1B_/*aac(6’)-Iaa*/*strA*/*strB*/*tet*(B)/*qnrS1*/*sul2*	AMP/CTX/STR/TET	N	ND	3,713
SZ22HS74*	*S*. 1,4,[5],12:i:-	34	F, 10m	2022.03.11	*bla*_CTX-M-55_	*bla*_TEM-1B_/*aac(6’)-Iaa*/*strA*/*strB*/*tet*(A)/*tet*(B)/*floR*/*qnrS1*/*sul2*/*dfrA14*	AMP/CTX/STR/TET/CHL/FFC/SXT	N	IncI1	84,050
SZ22HS76*	*S*. 1,4,[5],12:i:-	34	M, 34y	2022.04.03	*bla*_CTX-M-65_	*bla*_OXA-10_/*aac(6’)-Iaa*/*aph(3’)-Ia*/*aph(4)-Ia*/*aac(3)-IV*/*aadA1*/*aadA2*/*strA*/*strB*/ *tet*(A)/*tet*(B)/*floR*/*cmlA1*/*qnrS1*/*sul2*/*sul3*/*dfrA14*/*arr-2*/*lnu*(F)	AMP/CTX/GEN/STR/TET/CHL/FFC/SXT	N	IncHI2	164,862
SZ22HS98	*S*. Agona	13	F, 4y	2022.09.23	*bla*_CTX-M-55_	*aac(6’)-Iaa*/*aac(3)-IId*/*aadA22*/*strB*/*tet*(A)/*floR*/ *qnrS1*/*fosA7*/*sul3*/*lnu*(F)	AMP/CTX/GEN/STR/TET/CHL/FFC	N	ND	12,437
SZ22HS102	*S*. 1,4,[5],12:i:-	34	M, 24d	2022.10.27	*bla*_CTX-M-65_	*bla*_OXA-10_/*bla*_TEM-1B_/*aac(6’)-Iaa*/*aph(3’)-Ia*/*aph(4)-Ia*/*aac(3)-IV*/*aadA1*/*strA*/*strB*/*tet*(B)/*sul2*/*dfrA14*	AMP/CTX/GEN/STR/TET/SXT	N	ND	5,836

a , *indicates that strain could successfully transfer *bla*_CTX-M_/*bla*_CMY-2_ to *E. coli* C600 by conjugation; Underline indicates stool samples obtained from the same individual, SZ20HS5 and SZ20HS17, SZ20HS10 and SZ20HS18.

b F, female; M, male.

c AMP, ampicillin; CTX, cefotaxime; GEN, gentamicin; AMK, amikacin; STR, streptomycin; TET, tetracycline; CHL, chloramphenicol; NAL, nalidixic acid; CIP, ciprofloxacin; CL, colistin; FOS, fosfomycin; SXT, sulfamethoxazole/trimethoprim; all strains were susceptible to meropenem.

d N, not found.

e ND, not determined.

### Genetic structures of *bla*_CTX-M_/*bla*_CMY-2_ in eighteen cefotaxime-resistant *Salmonella* isolates

**Figure 4:**
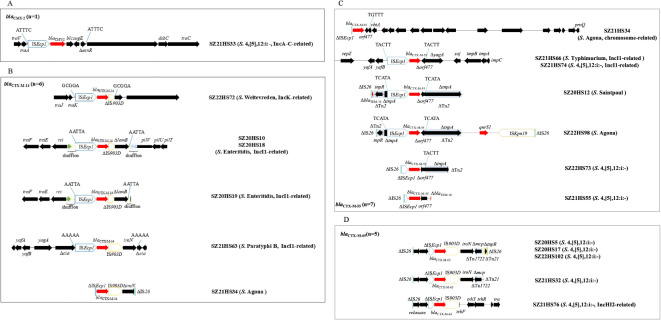
The genetic environments of *bla*_CTX-M_/*bla*_CMY-2_ in 18 *Salmonella* isolates in this study. **(A)** *bla*_CMY-2_; **(B)** *bla*_CTX-M-14_; **(C)** *bla*_CTX-M-55_; **(D)** *bla*_CTX-M-65_. The extents and directions of antibiotic resistance (red arrows) and other genes (black arrows) are indicated. ISs are shown as boxes labeled with their name. Δ indicates a truncated gene or mobile element. Arrows and sequences indicate direct repeats.

The lengths of *bla*_CTX-M_/*bla*_CMY-2_-carrying contigs ranged from 2,156 to 164,862 bp, and were located either on chromosome (n=1) or plasmids (n=9) (**Table 1**). Nine contigs were relatively short (2,156 to 12,437 bp) due to incomplete assembly and the presence of multiple insertion elements. These contigs lacked replicon genes or plasmid backbone, making it difficult to determine their exact location (**Table 1**; **Figure 4**).

In *S*. 4,[5],12:i:- isolate SZ21HS33, a 4,479-bp transposition unit (IS*Ecp1*-*bla*_CMY-2_-*blc*-*sugE*-Δ*ecnR*) was inserted downstream of the plasmid conjugal transfer gene *traA*, generating 5-bp direct repeats (DRs) (**Figure 4A**). This *bla*_CMY-2_-bearing contig (92,168 bp) was highly similar (>99.99%) to the corresponding region of IncA/C plasmids found in *Salmonella* isolates from China, such as pSa1753 (food, MT859309) and pR1041-Sal2-167k (hospital, OR095745) (**Supplementary Figure S1**). Additionally, seven additional resistance genes, including *bla*_OXA-10_, *addA1*, *aac(6’)-Ib3*, *aac(6’)-Ib-cr*, *qnrVC1*, *dfrA14*, and *mph*(A), were co-located within this *bla*_CMY-2_-carrying contig.

Six *Salmonella* isolates carried the *bla*_CTX-M-14_ gene. The *bla*_CTX-M-14_-positive contig (92,206-bp) from the *S*. Weltevreden isolate SZ22HS72 was similar to IncK1 plasmids from *E. coli*, such as pDETEC82 (patient, China, CP116171) and pF16EC0617-4 (human, South Korea, CP088378), with 85% coverage and 99.99% identity (**Supplementary Figure S1**). The *bla*_CTX-M_-_14_ gene was associated with the commonly observed 3,060-bp structure (IS*Ecp1*-*bla*_CTX-M_-_14_-ΔIS*903D*), inserted downstream of the plasmid conjugal transfer gene *trak* with 5-bp DRs (5’-GCGGA-3’) (**Figure 4B**). The *bla*_CTX-M-14_ gene was located on an IncI1 plasmid in four isolates (SZ20HS10, SZ20HS18, SZ20HS19, and SZ21HS63). Two *S*. Enteritidis isolates, SZ20HS10 and SZ20HS18, shared an identical *bla*_CTX-M-14_-positive contig (92,725-bp), showing 97% coverage and 99.97% identity with IncI1 plasmid pIncI1-CTX-M-14 (MN125610), obtained from a *S*. Enteritidis isolate from chicken in China (**Supplementary Figure S1**). A similar *bla*_CTX-M-14_-bearing contig (92,219-bp) was also found in *S*. Enteritidis isolate SZ20HS19 (**Supplementary Figure S1**). In these isolates, a 4,133-bp segment (IS*Ecp1*-*bla*_CTX-M-14-_ΔIS*903D-*Δ*lamB*) with 5-bp DRs (5’-AATTA-3’) was inserted into the shufflon region of the IncI1 plasmid (**Figure 4B**). In *S*. Paratyphi B isolate SZ21HS63, the *bla*_CTX-M-14_-carrying contig (96,781-bp) exhibited 100% similarity to IncI1 plasmid pSZB23-1 (CP107011) from a clinical *S*. Paratyphi B isolate in Shenzhen, China (**Supplementary Figure S1**). The typical transposition unit (IS*Ecp1*-*bla*_CTX-M-14_-IS*903D*-*iroN*), flanked by 5-bp DRs (5’-AAAAA-3’), was inserted into the *cia* gene, which encodes a colicin-like pore-forming protein (**Figure 4B**). A similar structure (ΔIS*Ecp1*-*bla*_CTX-M_-_14-_IS*903D*-Δ*iroN*) was observed in *S*. Agona isolate SZ21HS34, although IS*Ecp1* and *iroN* were incomplete.

The isolate SZ21HS34 also carried another *bla*_CTX-M_, *bla*_CTX-M-55_, located on the chromosome. The *bla*_CTX-M-55_-positive contig (22,529 bp) was identical to the corresponding region in *Salmonella* chromosomes, such as SSDFZ54 (CP034819) and SCFS (CP051218). Furthermore, the 1,266-bp segment (ΔIS*Ecp1*-*bla*_CTX-M-55_-Δ*orf477*) in SZ21HS34 was commonly found in plasmids and chromosomes of various species (e.g., *Klebsiella pneumoniae*, *E. coli*, and *S*. *enterica*). In SZ21HS66 (*S*. Typhimurium) and SZ22HS74 (*S*. 4,[5],12:i:-), *bla*_CTX-M-55_ was located on the IncI1 plasmid, which showed >99.99% similarity to IncI1 plasmids from *S.* Typhimurium strains in China, such as pST53-2 (patient, CP050747) and pS29-IncI1 (human, CP085700) (**Supplementary Figure S1**). The 2,971-bp transposition unit (IS*Ecp1*-*bla*_CTX-M-55_-Δ*orf477*) was inserted into the *yagA* gene, generating 5-bp DRs (5’-TACTT-3’) (**Figure 4C**). The identical 2,971-bp *bla*_CTX-M-55_ unit was also observed in *S*. Saintpaul isolate SZ20HS12, where it was inserted into the transposase gene *tnpA* of transposon Tn*2* (*bla*_TEM-1b_-*tnpR*-*tnpA*), although the β-lactam resistance gene *bla*_TEM-1b_ was truncated by an incomplete insertion sequence IS*26* (**Figure 4C**). An identical *bla*_CTX-M-55_-carrying structure was found in *S*. Agona isolate SZ22HS98, followed by a 5,752-bp segment (*qnrS1*-IS*Kpn19*-IS*26*) (**Figure 4C**). However, the incomplete transposon Tn*2* was truncated by IS*26* at the resolvase gene *tnpR* (**Figure 4C**). A 3,713-bp similar segment (ΔIS*26-*ΔIS*Ecp1*-*bla*_CTX-M-55_-Δ*orf477-*ΔTn*2*) was observed in SZ22HS73 (*S*. 4,[5],12:i:-), with IS*Ecp1* truncated by IS*26* at the 5’ end (**Figure 4C**). Similarly, a 2,156-bp segment was found in SZ21HS55 (*S*. 4,[5],12:i:-), including the commonly observed structure (ΔIS*26-*ΔIS*Ecp1*-*bla*_CTX-M-55_-*orf477*) and a truncated *bla*_TEM-1b_ downstream (**Figure 4C**).

Five *S*. 4,[5],12:i:- isolates carried *bla*_CTX-M-65_. As shown in **Figure 4D**, three isolates (SZ20HS5, SZ20HS17 and SZ22HS102) shared an identical *bla*_CTX-M-65_ region, including the typical transposition unit (ΔIS*Ecp1*-*bla*_CTX-M-65_-IS*903*-*iroN*), followed by three incomplete mobile elements (ΔTn*1722*, ΔTn*21*, and ΔIS*26*). A 1,205-bp region, containing two hypothetical proteins and an incomplete IS*26* (76-bp), was found upstream of the *bla*_CTX-M-65_ unit. A similar region with 277-bp deletions of ΔTn*21* and ΔIS*26* was identified in SZ21HS32. Similarly, a 3,645-bp *bla*_CTX-M-65_ region was observed in SZ21HS76, including the upstream 1,205-bp segment and the *bla*_CTX-M-65_ unit (ΔIS*Ecp1*-*bla*_CTX-M-65_-IS*903*). This region was followed by IncHI2 plasmid conjugal transfer genes, such as Δ*trhF*, *trhY*, *trhR*, and *traH*. The *bla*_CTX-M-65_-positive contig (164,862-bp) from isolate SZ21HS76 displayed >99.9% similarity to multiple IncHI2 plasmids, such as pS304_1 (*S*. Typhimurium, human, CP061127) and pHH194M-228K (*E. coli*, migratory bird, CP101516) (**Supplementary Figure S1**).

## Discussion

So far, among the more than 2,600 identified *Salmonella* serovars, many are known to cause human salmonellosis (EFSA and ECDC, 2023; Monte and Sellera, 2020). In this study, among the 96 *Salmonella* isolates representing 17 different serovars, the most prevalent was *S*. Typhimurium monophasic variant (*S*. 4,[5],12:i:-), accounting for 37.50%, followed by *S.* Enteritidis (15.63%) and *S.* Typhimurium (14.58%). This is consistent with previous study indicating that *S*. 4,[5],12:i:-, *S*. Enteritidis, and *S*. Typhimurium were the most common serovars among outpatients in Shaoxing, China (Chen et al., 2022). Similarly, an analysis of over 35,000 *S. enterica* strains from human and non-human sources across 23 Chinese provinces or municipal cities from 2006 to 2019 reported *S*. Typhimurium (including its monophasic variant) as the most common serovar, followed by *S*. Enteritidis (Wang et al., 2022a). In Europe, the three most commonly reported serovars since 2014 are *S*. Enteritidis, *S*. Typhimurium, and its monophasic variant *S*. 4,[5],12:i:-, which together account for more than 70% of human cases (EFSA and ECDC, 2023). *S.* Typhimurium has been identified as one of the most common serovars responsible for foodborne gastroenteritis worldwide (CDC, 2019; EFSA and ECDC, 2023; Nambiar et al., 2024; Van Puyvelde et al., 2023). Its monophasic variant, *S*. 4,[5],12:i:-, which lacks the ability to express the second-phase flagellar antigen, has become increasingly prevalent and is now a dominant serotype causing human salmonellosis (Wang et al., 2023a). In the United States, infections caused by *S*. 4,[5],12:i:- increased between 2009 and 2018, and this serotype has become the fifth most commonly reported serotype causing human illness (Plumb et al., 2023). Recently, an outbreak of multidrug-resistant *S*. 4,[5],12:i:- ST34 infection linked to chocolate products has been reported globally (Lund et al., 2022).

The widespread use of antimicrobial agents has led to an increase in MDR *Salmonella*. In this study, *Salmonella* isolates carried between one and 21 resistance genes, with 70.83% classified as MDR, severely limiting therapeutic options for clinical infections. Resistance to several important antibiotics was observed in our study. For instance, Fosfomycin exhibits strong antimicrobial activity against both Gram-negative and Gram-positive bacteria (Falagas et al., 2016). However, the widespread dissemination of fosfomycin resistance genes has led to an increase in reports of fosfomycin-resistant *Salmonella* (Aghamali et al., 2019; Fang et al., 2020). In Gram-negative bacteria, several plasmid-mediated fosfomycin resistance genes have been identified, with *fosA3* being the most prevalent in Enterobacteriaceae, including *Salmonella* (Aghamali et al., 2019; Fang et al., 2020). In this study, one *S*. 4,[5],12:i:- isolate carrying *fosA3* was found to be resistant to fosfomycin. Although *fosA7* was detected in three *Salmonella* isolates in our study, it is present as a silent gene located on the chromosomes of certain *Salmonella* serotypes, and does not contribute to fosfomycin resistance (Monte et al., 2023; Wang et al., 2024). The fosfomycin-resistant *S*. 4,[5],12:i:- isolate carried the *rmtB* gene, conferring resistance to gentamicin and amikacin. The 16S rRNA methylase genes mediate high-level resistance to aminoglycosides, with *armA* and *rmtB* being the most prevalent in various Gram-negative bacteria, including *Escherichia coli*, *Salmonella*, *K. pneumoniae*, and *Acinetobacter baumannii* (Doi et al., 2016). Resistance to colistin, the last-resort antibiotic for Gram-negative pathogens, was observed in four *S*. Enteritidis isolates in our study. However, neither the colistin resistance gene *mcr* nor mutations in PmrA/B-PhoP/Q were identified, suggesting that their resistance was probably intrinsic, due to the O-antigen epitope of group D *Salmonella* governing the levels of colistin susceptibility (Ricci et al., 2020).

Fluoroquinolones and third-generation cephalosporins, such as ciprofloxacin and cefotaxime, are first-line treatments for *Salmonella* infections (Ruiz et al., 2004). Fluoroquinolone resistance in Gram-negative bacteria is primarily associated with mutations in the chromosomal QRDRs of the *gyrA* and *parC* genes, as well as plasmid-mediated quinolone resistance (PMQR) genes (Hooper and Jacoby, 2015). In this study, mutations in the *gyrA* gene were identified in 21 *Salmonella* isolates, mainly at positions D87G/D87Y, with S83F also being detected. However, mutations in *gyrB*, *parC*, or *parE* were not identified in this study. GyrA position 87 is a common mutation site in *Salmonella*, and mutations at *gyrA* position 83 and *parC* position 80 are also frequently observed, whereas the frequency of *gyrB* and *parE* mutations is relatively low (Wang et al., 2022a). Additionally, 37 *Salmonella* strains harbored at least one PMQR gene in our study, with *qnrS1* being the most prevalent. Worryingly, a high prevalence (10.45%) of 1,962 *Salmonella* isolates in China carried the PMQR genes, with *qnrS1* being the most common (Wang et al., 2022a). As previously described (Bai et al., 2016; Wang et al., 2023b), the combination of chromosomal *gyrA* mutations and PMQR genes contribute to resistance to nalidixic acid and ciprofloxacin in *Salmonella*.

In this study, 18.75% of *Salmonella* isolates exhibited resistance to the third-generation cephalosporin cefotaxime, due to the presence of *bla*_CTX-M_, with one strain carrying *bla*_CMY-2_. Since the discovery of *bla*_CTX-M_, its prevalence has increased globally, making it one of the most common ESBL gene worldwide (Bevan et al., 2017). In China, *bla*_CTX-M_ is also the predominant ESBL gene in *Salmonella* strains from human, animal, and food sources (Bao et al., 2024; Wang et al., 2022a). High detection rates of *bla*_CTX-M_ have been observed in several *Salmonella* serotypes, such as *S.* Typhimurium, *S*. Kentucky, and *S*. Indiana (Sun et al., 2022; Wang et al., 2020, 2023b). Currently, more than 260 different subtypes of *bla*_CTX-M_ have been identified globally (https://www.ncbi.nlm.nih.gov/pathogens/refgene/), with *bla*_CTX-M-14_ and *bla*_CTX-M-15_ being the most prevalent subtypes globally, and *bla*_CTX-M-55_ being predominant mainly in Asia such as China (Bevan et al., 2017). In this study, three *bla*_CTX-M_ subtypes were identified: *bla*_CTX-M-55_ (38.89%), *bla*_CTX-M-14_ (33.33%), and *bla*_CTX-M-65_ (27.78%). In a previous study, 2,283 *Salmonella* strains from human feces and animal-derived food samples (chicken, pork, and seafood) collected across five provinces in China were analyzed, 200 of them were positive for *bla*_CTX-M_ with *bla*_CTX-M-65_, *bla*_CTX-M-123_ and *bla*_CTX-M-14_ being the most prevalent (Wang et al., 2020). Similarly, Wang et al. (2022a) performed whole-genome sequencing on 1,962 *Salmonella* isolates (from both human and non-human sources) across 22 provinces and municipalities in China, and identified 13 distinct *bla*_CTX-M_ subtypes, including *bla*_CTX-M-55_, *bla*_CTX-M-14_, and *bla*_CTX-M-65_. Interestingly, despite the global dominance of *bla*_CTX-M-15_, it was not detected in our study, reflecting its lower prevalence in *Salmonella* from China (Bevan et al., 2017; Jiang et al., 2022; Wang et al., 2022a). Although co-resistance to both cephalosporins and fluoroquinolones in *Salmonella* has been increasingly reported (Bai et al., 2016; Liu et al., 2023; Nambiar et al., 2024; Wang et al., 2023b), only one *S*. 4,[5],12:i:- isolate in our study exhibited co-resistance to cefotaxime and ciprofloxacin.

The global dissemination of *bla*_CTX-M_ is partly driven by some successful clones. For example, *E. coli* ST131, which produces CTX-M-15, is a high-risk international clone, particularly in hospitals (Becerra-Aparicio et al., 2023; Bevan et al., 2017; Nicolas-Chanoine et al., 2014). Similarly, *S*. 4,[5],12:i:- ST34 and *S*. Kentucky ST198 have disseminated globally, facilitating the spread of resistance genes such as *bla*_CTX-M-65_, *bla*_CTX-M-55_, and *bla*_CTX-M-14_ (Hawkey et al., 2019; Protonotariou et al., 2022; Wang et al., 2023b). Notably, two *S*. 4,[5],12:i:- isolates and two *S*. Enteritidis isolates in this study, obtained from the same infants at different times, shared identical STs, resistance profiles, resistance genes, mutations, and *bla*_CTX-M_-carrying contigs. This suggests long-term colonization of ESBL-producing *S*. 4,[5],12:i:- or *S*. Enteritidis clones in individual patients for periods exceeding three or six weeks, likely contributing to the persistence and transmission of antimicrobial resistance within the local population. Similar long-term persistence of bacterial clones has been previously reported, such as the *S*. Kentucky ST198 clone in a patient with inflammatory bowel disease (Jiang et al., 2024), and CTX-M-producing *E. coli* in healthy food handlers for durations ranging from three months to two years (Nakane et al., 2016). Many bacterial species can establish persistent infections in their hosts, even after antibiotic treatment, due to factors such as host immunocompromise, bacterial immune evasion, and/or inadequate eradication of the pathogen by antibiotics (Fisher et al., 2017). Given that antimicrobial drugs are sometime ineffective in eliminating long-term colonization, it is crucial to explore alternative strategies, such as phage therapy, for bacterial decolonization in humans to prevent infections and reduce the spread of MDR organisms (Fang et al., 2024).

Pandemic plasmids play a significant role in the global transmission of *bla*_CTX-M_ in *Enterobacteriaceae*, with plasmids such as IncI1, IncK1, IncHI2, IncF, and IncN acting as key vectors for horizontal gene transfer (Bevan et al., 2017; Rozwandowicz et al., 2018). The IncI1 plasmid, one of the most common types in *Enterobacteriaceae* from humans, animals and the environment, is an important carrier of *bla*_CTX-M_ genes (Carattoli et al., 2021; Rozwandowicz et al., 2018). IncI1 plasmids are linked to the global spread of multiple *bla*_CTX-M_ variants, such as *bla*_CTX-M-14_ and *bla*_CTX-M-55_ in this study, as well as *bla*_CTX-M-1_, *bla*_CTX-M-3_, *bla*_CTX-M-15_, and *bla*_CTX-M-101_ (Irrgang et al., 2018; Protonotariou et al., 2022; Qin and Zhang, 2023; Saidani et al., 2019; Yu et al., 2024). Other plasmids, such as IncK1, are also crucial for the dissemination of *bla*_CTX-M_ in *Enterobacteriaces* from diverse sources. For example, IncK1 plasmids are common vectors for horizontal transfer of *bla*_CTX-M-14_ in *E. coli* isolates from both humans and animals in Europe (Rozwandowicz et al., 2017; Stokes et al., 2012; Valverde et al., 2009) and in *E. coli* isolates from healthy volunteers in Yangzhou, China (Wang et al., 2022b). Similarly, IncHI2 plasmids, which are prevalent in *Salmonella* isolates and often associated with MDR (Chen et al., 2016; McMillan et al., 2020), are important carriers of *bla*_CTX-M-14/-55/-65_ in *E*. *coli* and *Salmonella* isolates from humans, animals, and food products in China (Jiang et al., 2022; Li et al., 2022; Wang et al., 2018; 2022b; Tian et al., 2022).

The IncA/C plasmid has also emerged as a major vector for resistance genes, particularly in the spread of cephalosporinase genes like *bla*_CMY-2_ (Rozwandowicz et al., 2018). IncA/C plasmids carrying *bla*_CMY-2_ have been frequently detected in *E. coli* and *Salmonella* isolates from both animals and humans (He et al., 2021; Liu et al., 2023; Zhang et al., 2024; Zheng et al., 2022). In this study, one *S*. 4,[5],12:i:- isolate carried *bla*_CMY-2_ on an IncA/C plasmid highly similar to other IncA/C plasmids from *Salmonella*. In addition to *bla*_CMY-2_, IncA/C plasmids have been described to mediate the spread of ESBL genes (e.g., *bla*_CTX-M_) and carbapenemase genes (e.g., *bla*_NDM-1_) in *E. coli*, *Salmonella*, and *K. pneumoniae* isolates from various sources (Papa-Ezdra et al., 2021; Tello et al., 2022; Villa et al., 2015; Wailan et al., 2016; Wasyl et al., 2015).

Insertion sequence (IS) facilitate the horizontal transfer of *bla*_CTX-M_ genes between plasmids and chromosomes, with IS*Ecp1* and IS*26* being key elements in this process (Castanheira et al., 2021; Partridge et al., 2018). IS*Ecp1*, a member of the IS*1380* family, transposes by recognizing the right inverted repeat sequence or its similar sequence and can carry adjacent structures including drug resistance genes, often generating 5-bp DRs (Partridge et al., 2018). In this study, IS*Ecp1* mediated the transfer of *bla*_CMY-2_ and *bla*_CTX-M_ genes. IS*Ecp1* typically locates upstream of *bla*_CTX-M_, providing a promoter for its expression (Partridge et al., 2018; Poirel et al., 2003). Interestingly, the *bla*_CTX-M-55_ gene was found on the chromosome of a *S*. Agona isolate, associated with an incomplete IS*Ecp1* insertion upstream. Although *bla*_CTX-M_ is frequently plasmid-borne, there is increasing evidence of its integration into chromosomal DNA, mediated by mobile elements, in *E. coli*, *Salmonella*, and *K. pneumoniae* isolates (Huang et al., 2017; Jiang et al., 2023; Shen et al., 2016; Yoon et al., 2020). For instance, IS*Ecp1*-mediated *bla*_CTX-M-14_ integration into the type VI secretion system on the chromosome of *S*. Kentucky ST198.2-1 clade has been reported in Europe and China (Hawkey et al., 2019; Wang et al., 2023b). Once integrated into the chromosome, resistance genes such as *bla*_CTX-M_ can be vertically transmitted to daughter cells, becoming intrinsic resistance determinants. Furthermore, various mobile elements are involved in the acquisition and spread of *bla*_CTX-M_ and *bla*_CMY_ genes, such as the class I integron-IS*CR1* complex (*bla*_CTX-M-2_ and *bla*_CTX-M-9_) and IS*1294* (*bla*_CMY-2_) (Castanheira et al., 2021; Tagg et al., 2014).

However, our study has several limitations. Our study provides limited information and insights into Salmonellosis surveillance, since the analysis was based on only 96 *Salmonella* isolates collected from a single hospital in China. The small sample size and restricted geographic scope may not adequately represent the overall epidemiology of Salmonellosis or the prevalence of antimicrobial resistance in this region. Furthermore, although 17 serotypes were identified, the limited number of isolates for each serotype may have resulted in the underrepresentation of other important serotypes. The small sample size per serotype also hinders comparisons of resistance patterns across different serotypes. Therefore, continuous and expanded surveillance of Salmonellosis involving multiple hospitals and a larger number of samples, is essential to provide a comprehensive understanding of its epidemiology and antimicrobial resistance patterns.

In conclusion, *S*. 4,[5],12:i:-, *S*. Enteritidis, and *S*. Typhimurium are the predominant serotypes in this clinical setting, exhibiting a concerning prevalence of MDR isolates. The dissemination of *bla*_CTX-M_/*bla*_CMY-2_ among clinical *Salmonella* isolates is primarily mediated through horizontal gene transfer facilitated by global successful pandemic plasmids (e.g., IncI1) and associated mobile elements (IS*Ecp1*). The findings highlight the urgent need for implementation of targeted antimicrobial stewardship programs, reinforcement of infection control measures, and the development of alternative therapeutic strategies, such as phage therapy, to manage persistent infections and curb the spread of MDR strains. Long-term colonization and plasmid-mediated resistance spread reveal gaps in our understanding of transmission dynamics, underscoring the necessity of incorporating molecular surveillance into routine clinical practice to enhance prevention and control measures.

## Data Availability

The datasets presented in this study can be found in online repositories. The names of the repository/repositories and accession number(s) can be found in the article/**Supplementary Material**.
