# Supplementary material for: Stool carriage of CTX-M/CMY-producing Salmonella enterica in a Chinese tertiary hospital in Shenzhen, China
Source: Front Cell Infect Microbiol. 2025 Mar 13;15:1544757. doi: 10.3389/fcimb.2025.1544757 (PMC11966408; doi:10.3389/fcimb.2025.1544757)
Supplement: Supplementary file 1 [file Table1.docx]

Table S1. Phenotypic antimicrobial resistance patterns for *Salmonella* serotypes

| **patterns** | **number** |
| --- | --- |
| pansusceptible | 7 |
| TET | 4 |
| NAL | 7 |
| AMP/CTX | 2 |
| AMP/STR | 2 |
| AMP/NAL | 1 |
| STR/TET | 2 |
| TET/CHL | 2 |
| AMP/GEN/STR | 1 |
| AMP/STR/TET | 12 |
| AMP/STR/NAL | 2 |
| AMP/TET/SXT | 4 |
| AMP/CHL/SXT | 2 |
| AMP/NAL/SXT | 1 |
| STR/TET/NAL | 1 |
| AMP/CTX/STR/TET | 1 |
| AMP/STR/NAL/CL | 2 |
| AMP/TET/CHL/SXT | 13 |
| STR/TET/CHL/SXT | 1 |
| AMP/CTX/STR/TET/NAL | 1 |
| AMP/CTX/TET/CHL | 1 |
| AMP/CTX/TET/CHL/SXT | 1 |
| AMP/STR/TET/CHL/SXT | 4 |
| STR/TET/CHL/NAL/SXT | 2 |
| AMP/CTX/STR/TET/NAL/CL | 2 |
| AMP/CTX/STR/TET/CHL/SXT | 4 |
| AMP/GEN/TET/CHL/NAL/SXT | 2 |
| AMP/CTX/GEN/STR/AMI/TET/FOS | 1 |
| AMP/CTX/GEN/STR/TET/CHL | 1 |
| AMP/CTX/GEN/STR/TET/SXT | 1 |
| AMP/CTX/GEN/STR/TET/CHL/SXT | 2 |
| AMP/GEN/STR/TET/CHL/NAL/SXT | 4 |
| AMP/GEN/TET/CHL/NAL/CIP | 1 |
| AMP/GEN/TET/CHL/NAL/CIP/SXT | 1 |
| AMP/CTX/GEN/STR/TET/CHL/NAL/CIP/SXT | 1 |

**Figure S1**. Linear sequence comparison of *bla*_CTX-M_/*bla*_CMY_-carrying contig (plasmid-related) with other similar plasmids using Easyfig.
