# Supplementary material for: Stool carriage of CTX-M/CMY-producing Salmonella enterica in a Chinese tertiary hospital in Shenzhen, China
Source: Front Cell Infect Microbiol. 2025 Mar 13;15:1544757. doi: 10.3389/fcimb.2025.1544757 (PMC11966408; doi:10.3389/fcimb.2025.1544757)
Supplement: Supplementary file 2 [file Presentation1.pptx]

## Slide 1
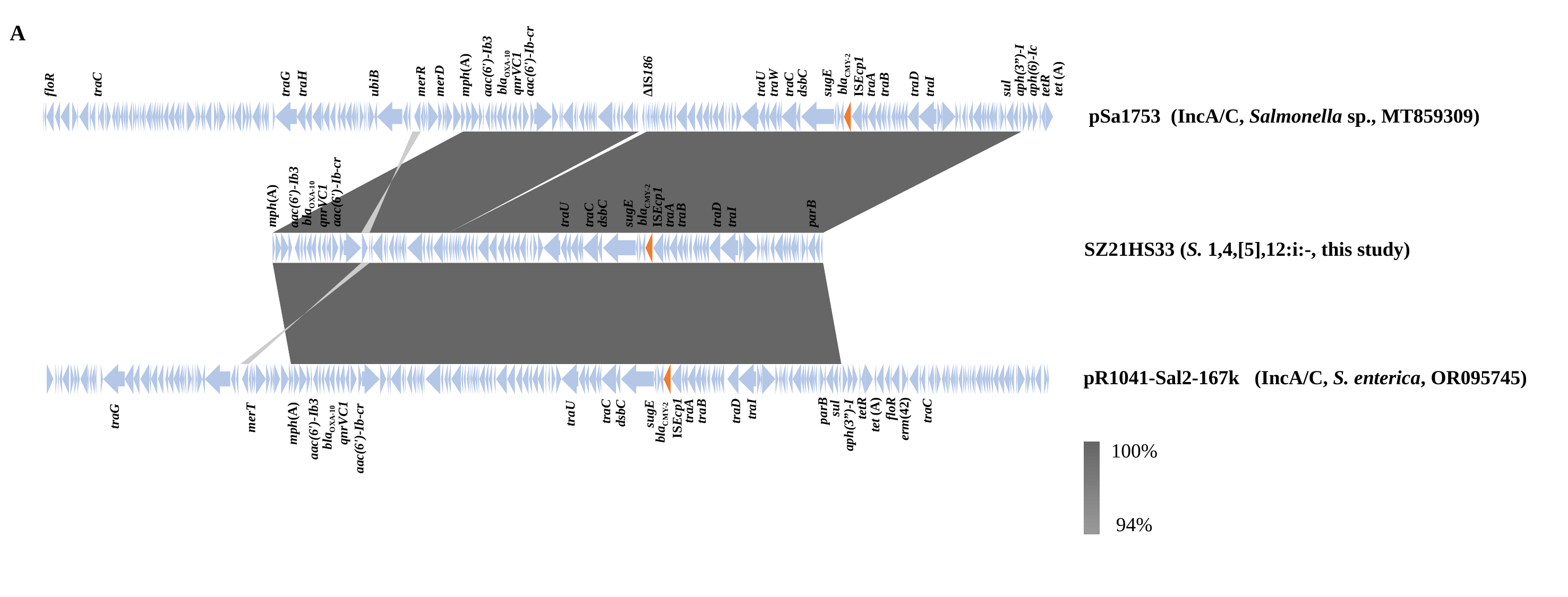

A
aac(6')-Ib-cr
aac(6')-Ib3
aph(3”)-I
aph(6)-Ic
blaOXA-10
qnrVC1
blaCMY-2
mph(A)
ISEcp1
ΔIS186
tet (A)
merD
merR
sugE
traW
dsbC
ubiB
traH
traG
traU
traD
traC
traC
traA
traB
floR
tetR
traI
sul
pSa1753 (IncA/C, Salmonella sp., MT859309)
aac(6')-Ib-cr
aac(6')-Ib3
blaOXA-10
blaCMY-2
qnrVC1
mph(A)
ISEcp1
sugE
dsbC
parB
traU
traD
traC
traA
traB
traI
SZ21HS33 (S. 1,4,[5],12:i:-, this study)
pR1041-Sal2-167k (IncA/C, S. enterica, OR095745)
tetR
sul
floR
traI
parB
traA
traC
traD
traB
traC
dsbC
traU
sugE
tet (A)
traG
merT
ISEcp1
erm(42)
blaCMY-2
qnrVC1
mph(A)
aph(3”)-I
blaOXA-10
aac(6')-Ib3
aac(6')-Ib-cr
100%
94%

## Slide 2
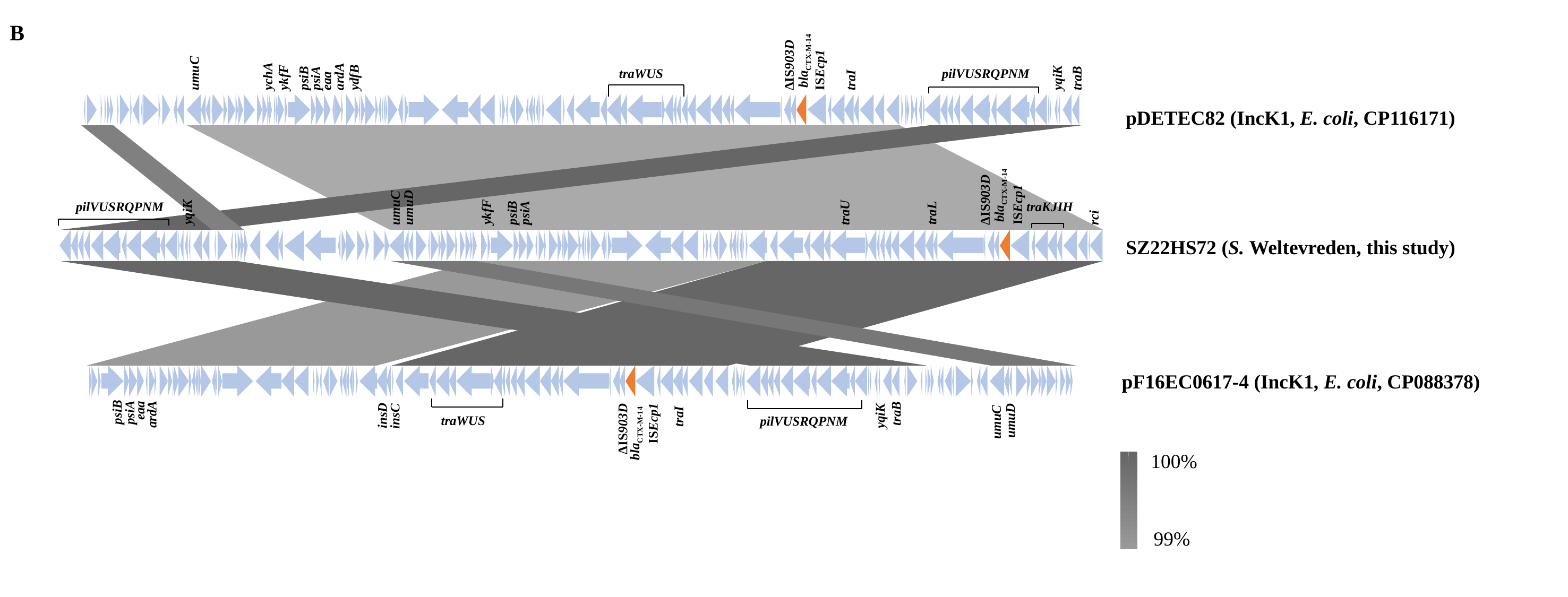

B
blaCTX-M-14
ΔIS903D
ISEcp1
traWUS
pilVUSRQPNM
umuC
ychA
ardA
ykfF
ydfB
yqiK
psiB
psiA
traB
traI
eaa
pDETEC82 (IncK1, E. coli, CP116171)
blaCTX-M-14
ΔIS903D
ISEcp1
pilVUSRQPNM
traKJIH
umuD
umuC
ykfF
yqiK
traU
psiB
psiA
traL
rci
SZ22HS72 (S. Weltevreden, this study)
pF16EC0617-4 (IncK1, E. coli, CP088378)
eaa
psiB
psiA
traB
ardA
insD
yqiK
traI
insC
traWUS
umuD
pilVUSRQPNM
umuC
ISEcp1
ΔIS903D
blaCTX-M-14
100%
99%

## Slide 3
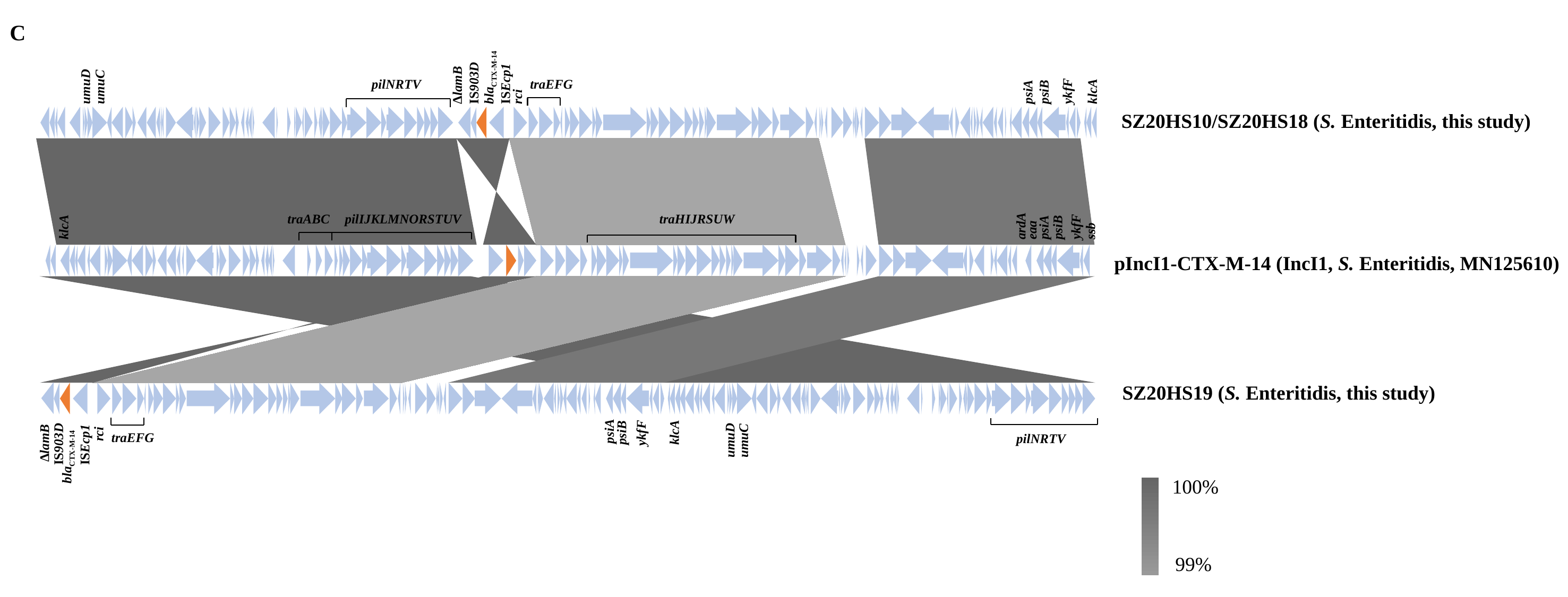

C
blaCTX-M-14
psiA
ΔlamB
IS903D
psiB
ykfF
klcA
umuD
ISEcp1
pilNRTV
umuC
traEFG
rci
SZ20HS10/SZ20HS18 (S. Enteritidis, this study)
psiA
ardA
psiB
traABC
pilIJKLMNORSTUV
traHIJRSUW
ssb
ykfF
eaa
klcA
pIncI1-CTX-M-14 (IncI1, S. Enteritidis, MN125610)
SZ20HS19 (S. Enteritidis, this study)
psiA
rci
psiB
ykfF
klcA
ΔlamB
ISEcp1
IS903D
umuC
umuD
traEFG
pilNRTV
blaCTX-M-14
100%
99%

## Slide 4
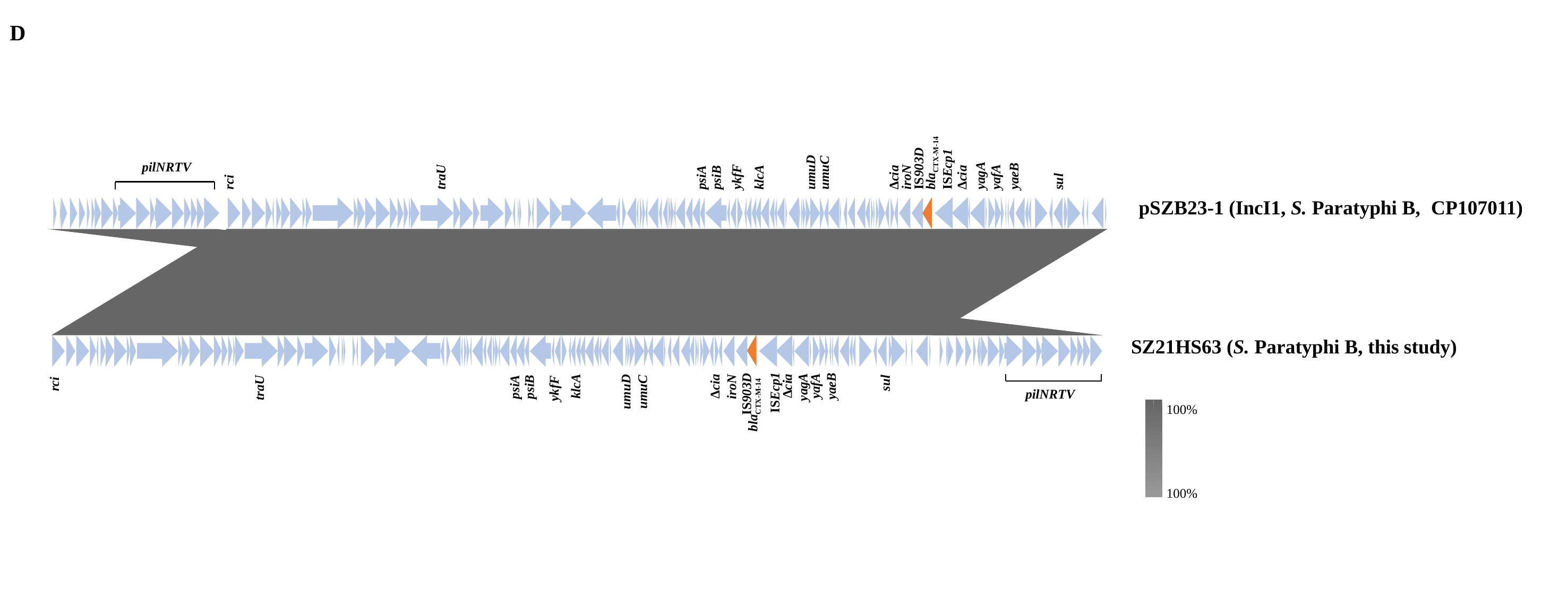

D
blaCTX-M-14
pilNRTV
IS903D
ISEcp1
umuD
umuC
yagA
yaeB
ykfF
yafA
klcA
traU
iroN
Δcia
Δcia
psiA
psiB
sul
rci
sul
rci
yafA
Δcia
Δcia
yaeB
klcA
iroN
psiA
psiB
yagA
traU
ykfF
umuC
umuD
ISEcp1
IS903D
pilNRTV
blaCTX-M-14
pSZB23-1 (IncI1, S. Paratyphi B,  CP107011)
SZ21HS63 (S. Paratyphi B, this study)
100%
100%

## Slide 5
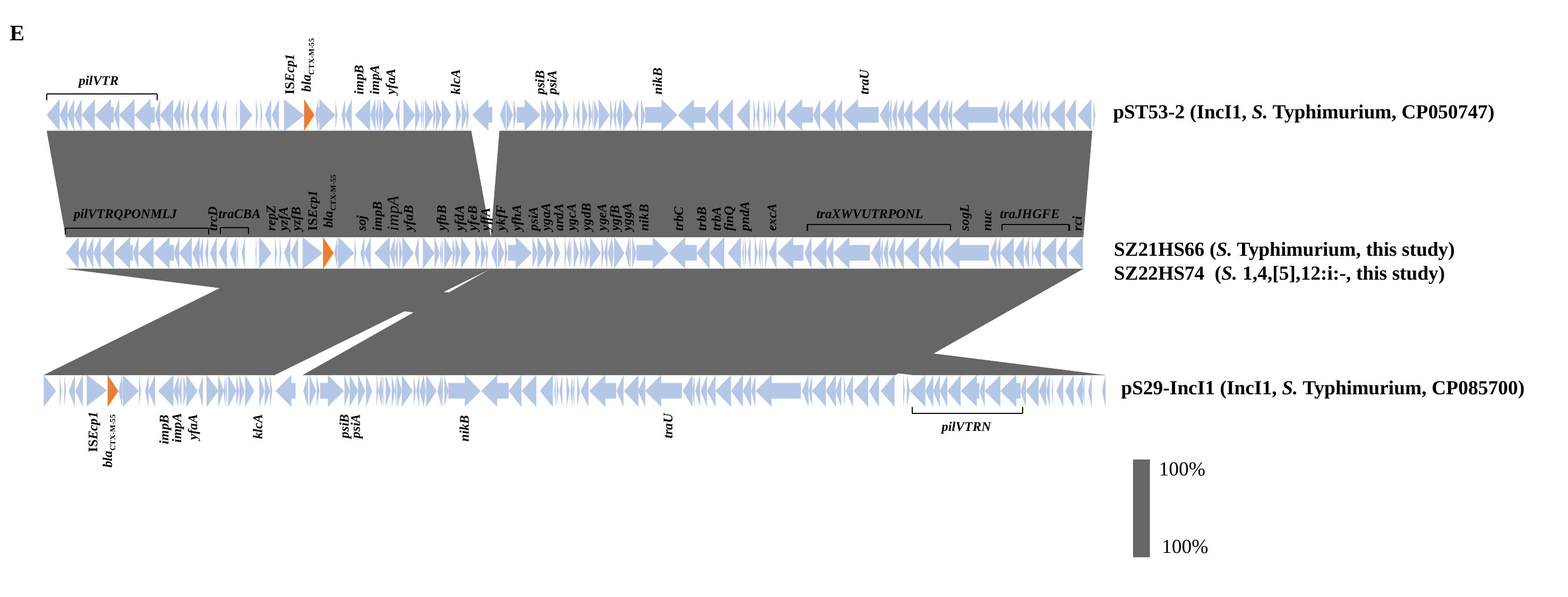

E
blaCTX-M-55
ISEcp1
impB
impA
pilVTR
nikB
yfaA
klcA
traU
psiB
psiA
pST53-2 (IncI1, S. Typhimurium, CP050747)
blaCTX-M-55
ISEcp1
impA
pilVTRQPONMLJ
traCBA
traXWVUTRPONL
traJHGFE
impB
pndA
ygaA
ygdB
yggA
ygcA
ygeA
excA
ardA
yfhA
nikB
sogL
repZ
yfaB
yfbB
yfdA
ykfF
ygfB
yfeB
finQ
trcD
yzfA
yzfB
psiA
trbC
trbB
trbA
yffA
nuc
soj
rci
SZ21HS66 (S. Typhimurium, this study)
SZ22HS74 (S. 1,4,[5],12:i:-, this study)
pS29-IncI1 (IncI1, S. Typhimurium, CP085700)
pilVTRN
traU
psiA
psiB
klcA
yfaA
impA
nikB
impB
ISEcp1
blaCTX-M-55
100%
100%

## Slide 6
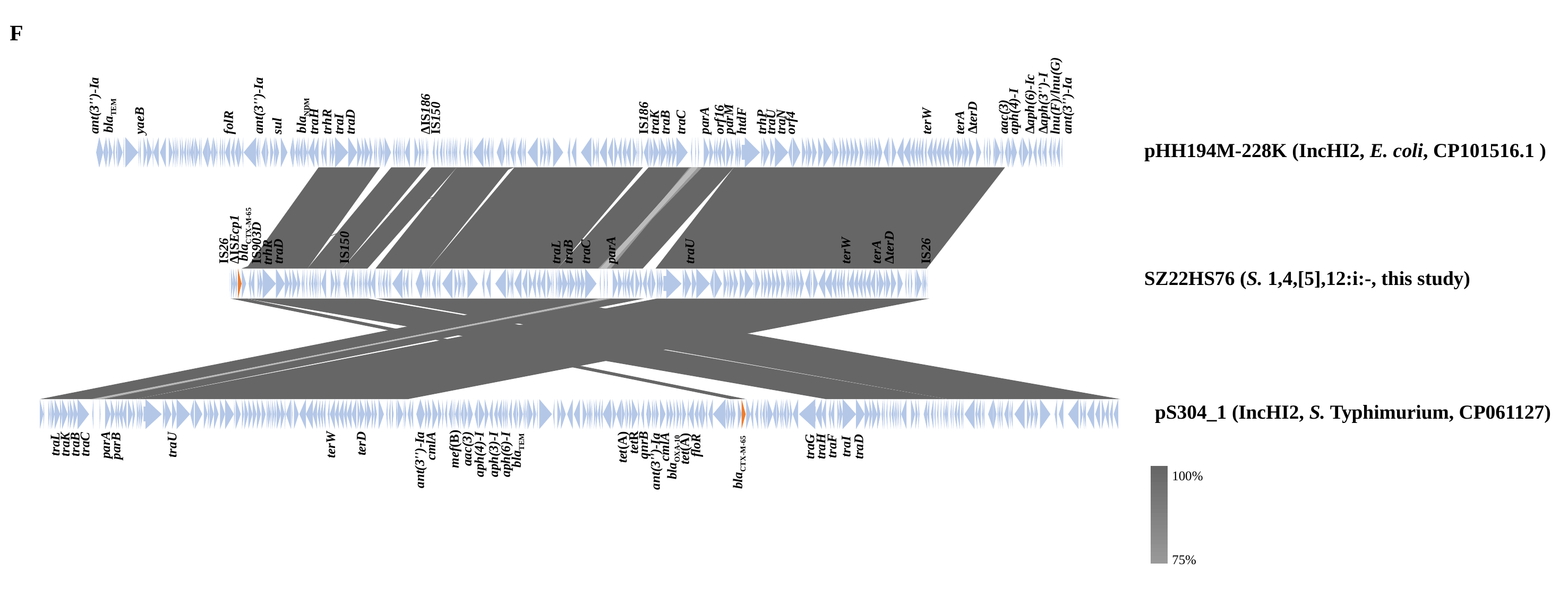

F
lnu(F)/lnu(G)
Δaph(3'')-I
Δaph(6)-Ic
ant(3'')-Ia
ant(3'')-Ia
aph(4)-I
ΔIS186
blaNDM
blaTEM
aac(3)
ΔterD
IS150
IS186
parM
orf16
yaeB
parA
htdF
terW
traH
trhR
traD
traU
traN
traK
traB
traC
trhP
folR
terA
orf4
traI
sul
blaCTX-M-65
ΔISEcp1
IS903D
ΔterD
IS150
parA
terW
IS26
IS26
traD
traU
traB
traC
traL
terA
trhR
ant(3'')-Ia
pHH194M-228K (IncHI2, E. coli, CP101516.1 )
SZ22HS76 (S. 1,4,[5],12:i:-, this study)
pS304_1 (IncHI2, S. Typhimurium, CP061127)
tetR
terD
traL
traK
traB
traC
terW
traU
qnrB
parA
floR
cmlA
traF
parB
traI
traH
traG
cmlA
traD
tet(A)
tet(A)
aac(3)
mef(B)
blaTEM
aph(4)-I
aph(3)-I
aph(6)-I
blaOXA-10
ant(3'')-Ia
ant(3'')-Ia
blaCTX-M-65
100%
75%
